# THE USE OF VIRTUAL VOLUNTEERISM WITHIN THE AMERICORPS SENIORS RSVP PROGRAM: IMPLICATIONS FOR PRACTICE

**DOI:** 10.1093/geroni/igae098.0829

**Published:** 2024-12-31

**Authors:** Jennifer Crittenden, Nathan Tarbox, Rachel Coleman, Leah Campbell, Adrienna Thorne, Chelsea Chapman

**Affiliations:** University of Maine, Orono, Maine, United States; University of Maine, Bangor, Maine, United States; University of Maine, Bangor, Maine, United States; University of Maine, Bangor, Maine, United States; University of Maine, Bangor, Maine, United States; University of Maine, Bangor, Maine, United States

## Abstract

The COVID-19 pandemic spurred the opportunity, and in some cases the imperative, to develop virtual volunteer (VV) options for older adults. The AmeriCorps Seniors RSVP program, one of the largest networks of older volunteers in the U.S., engages adults 55+ in a variety of local volunteer service opportunities. RSVP program directors were surveyed in 2023 (N = 138) to explore the use of VV, challenges associated with VV, and the opportunities for expansion of VV strategies with older adult volunteers. The most frequently cited VV assignments used by programs included: Community health education (53.5%, n = 23), financial services such as tax preparation and financial education (50%, n = 21), and companionship/friendly visiting (48.9%, n = 22). Common challenges cited by programs included a desire to return to in-person activities by older volunteers and beneficiaries, a lack of comfort with technology, lack of internet connection, and lack of funding resources to develop VV assignments. Statistical testing confirmed that the use of virtual strategies is not evenly distributed across geographical services areas with current (44.7%, n = 21) and past VV programs (44.4%, n = 8) more likely to serve metropolitan areas than rural or suburban service areas (X2 (2, N = 138) = 10.37, p <.01). Despite mixed levels of satisfaction with VV, approximately one in three programs (31.7%, n = 20) projected an expansion of VV with older volunteers into the future. Additional findings and associated programmatic considerations will also be discussed.

